# Preclinical Antileukemia Activity of Tramesan: A Newly Identified Bioactive Fungal Metabolite

**DOI:** 10.1155/2017/5061639

**Published:** 2017-11-15

**Authors:** M. R. Ricciardi, R. Licchetta, S. Mirabilii, M. Scarpari, A. Parroni, A. A. Fabbri, P. Cescutti, M. Reverberi, C. Fanelli, A. Tafuri

**Affiliations:** ^1^Department of Clinical and Molecular Medicine, Sapienza University of Rome, Rome, Italy; ^2^Department of Plant Biology, Sapienza University of Rome, Rome, Italy; ^3^Department of Life Sciences, University of Trieste, Trieste, Italy

## Abstract

Despite improvements that occurred in the last decades in the acute myeloid leukemia (AML) treatment, clinical results are still unsatisfactory. More effective therapies are required, and innovative approaches are ongoing, including the discovery of novel antileukemia natural compounds. Several studies have described the activity of extracts from mushrooms which produce compounds that exhibited immunological and antitumor activities. The latter has been demonstrated to be promoted in vitro by mushroom polysaccharides via induction of apoptosis. However, the antileukemia activity of these compounds on primary cells is still not reported. In the present study, we examined the in vitro effects of Tramesan (TR), a bioactive compound extracted from *Trametes versicolor*, on leukemic cell lines and primary cells. Our results demonstrated that TR induced a marked growth inhibition of leukemic cell lines and primary cells from AML patients. The antiproliferative effects of TR were associated in primary AML cells with a significant increase of apoptosis. No significant cytotoxic effects were observed in normal peripheral blood mononuclear cells (MNC) from healthy donors. Our data demonstrated a cytotoxic activity of TR on leukemia cells prompting further translational applications. Ongoing studies are elucidating the molecular mechanisms underlying its antileukemic activity.

## 1. Introduction

Acute leukemia is a disorder of the hematopoietic system characterized by clonal proliferation of variably differentiated myeloid or lymphoid precursors [1]. These hematological disorders show a high level of genetic complexity, which is the major explanation of the high rate of chemotherapy failure and of the result stagnation over the last decades [1]. Therefore, efforts have been made to identify novel therapeutic approaches aimed at overcoming drug resistance and exploring novel approaches based on chemo-free strategies. The successful example of the acute promyelocytic leukemia, nowadays treated in the majority of the cases without chemotherapy by regimen based on the combined use of retinoic acid and arsenic trioxide [2], is worth mentioning. On this basis, many attempts are ongoing to identify other similar approaches. In fact, besides targeted therapies active on leukemia-specific aberrant molecular alterations [3], an increasing number of studies are directed toward the drug discovery attempt, including the evaluation of natural compounds on different pathologies [4–6].

Mushrooms are rich sources of natural compounds used as biological response modifiers for a long time in oriental medicine and nowadays also in Western countries [7–9]. Mushrooms have emerged as a rich font of antioxidant, immunomodulating, anti-inflammatory, antimicrobial, and anticancer [10] compounds. Among different extracts from mushrooms, polysaccharides are important bioactive molecules and known as potent inhibitors of proliferation and apoptosis inducers in *in vitro* experimental models. In particular, the antitumor activity of PSK, a protein-bound polysaccharide obtained from the fungus *Trametes versicolor* (*T. versicolor*), has been documented in *in vitro* and *in vivo* experimental models [11]. Efficacy of the adjuvant immunotherapy with PSK was also demonstrated in human clinical trials [12–14]. Moreover, the combination of PSK with chemotherapy or radiotherapy has been shown to increase the efficacy of the latter in solid cancer treatments [7].

To date, few studies have reported the antineoplastic activity of PSK in hematologic models [11, 15–17]. In leukemia cell line, it was demonstrated that PSK exerted an antiproliferative and proapoptotic activity in HL-60 myeloid cell line, via activation of the mitochondrial and the p38 MAPK signaling cascades [16, 17].

Previous studies from our group have demonstrated that nonpurified extracts from the filtrates of *L. edodes* and *T. versicolor* inhibited mycotoxin synthesis in different fungi [18–20]. The mechanism of inhibition seems related to the promotion of fungal antioxidant activity on counteracting redox unbalance. It is now established that mycotoxigenic fungi are able to vehiculate the unscavenged ROS toward toxin synthesis.

Our group has patented a polysaccharidic fraction, *Tramesan* (TR), isolated from the liquid culture of the edible basidiomycete *T. versicolor* (Patent number RM2012A000573), able to significantly inhibit the synthesis of carcinogenic mycotoxins produced from different fungi (such as *Aspergillus flavus*, *A. parasiticus*, and Fusarium), by promoting an antioxidant activity [18, 20, 21].

The aim of this study was to investigate the activity of TR on leukemia cells by assessing proliferation inhibition and apoptosis induction on leukemia cell lines and on primary acute myeloid leukemia (AML) cells.

## 2. Materials and Methods

### 2.1. Fungal Strain and Growth Culture Conditions

The basidiomycete *Trametes versicolor* CF 117 was supplied from the mushroom collection of the laboratory of Mycology and Plant Pathology directed from Professor Corrado Fanelli (Sapienza University of Rome). The isolates were kept in potato dextrose agar (PDA) medium at 4°C, and the cultures have been renewed every 30 days.

### 2.2. Fungal Growth Substrate

*T. versicolor* was grown for 7 days in potato dextrose broth (PDB) and incubated at 25°C. The liquid culture was homogenized, in sterile condition in a Waring blender 8012. After homogenization, 5% (*v*/*v*) of the fungal culture was subsequently inoculated in 500 mL of PDB in 1 L Erlenmeyer flask and the *T. versicolor* cultures were incubated for 14 days at 25°C under shaken conditions (100 rpm). The mycelia were then separated from the culture filtrates by subsequent filtrations with different size filters (Whatman) to eliminate all the mycelia. The obtained culture filtrate was lyophilized and utilized for subsequent analyses.

### 2.3. Exopolysaccharide Extraction and Purification

The lyophilized *T. versicolor* culture filtrate (1 g) was dissolved in 30 mL of ultrapure H_2_O and filtrated to separate the insoluble from the soluble part. The sample was then incubated at 4°C for 2 h, and at the same time, a solution of absolute ethanol was cooled at 4°C. The cooled culture filtrate was added slowly to cooled ethanol, mixing with a glass rod. The precipitate was recovered by centrifugation at 4000 rpm for 20 min at 4°C. The recovered pellet was resuspended in 4 mL of ultrapure H_2_O and 4 mL of 20 mM phosphate buffer pH 7.5 to achieve the optimal conditions of ionic strength and pH for pronase E (Sigma-Aldrich) activity. The proteolysis was carried out at 37°C overnight. The elimination of salts and amino acids was performed through dialysis versus H_2_O, utilizing membrane of 12000 Da cutoff. The dialyzed samples were lyophilized and subjected to total carbohydrate determination by Yemm and Willis [22].

To separate, on the basis of their molecular weight, the exopolysaccharides precipitated with EtOH and present in *T. versicolor* culture filtrates, a size exclusion chromatography was performed. The mobile phase was NaNO_3_ 0.05 M degassed for about 30 min and filtered twice with Millipore 0.45 *μ*m filters. The Sephacryl S-300 (1.6 id × 90 cm) column was equilibrated with the eluent for about 30 h, with a flux of about 6 mL/h. The gel performances of the column allow to discriminate polysaccharides with a molecular weight between 2 and 400 kDa. About 40 mg of the sample was dissolved in 1.9 mL of 50 mM NaNO_3_, the solution was centrifuged for 10 min at 13000 rpm, and subsequently it was loaded in a column. The column was connected to a refractive index detector. Each fraction was collected every 20 min during 30 h.

The chromatographic fraction containing only polysaccharides (TR) was used for biological activity assays.

The polysaccharide TR, isolated and purified was patented from our group (Patent number RM2012A00057).

### 2.4. Cell Lines and Primary Samples

Human myeloid (OCI-AML3) and lymphoid (Jurkat) cell lines were maintained in RPMI 1640 medium containing 10% heat-inactivated fetal calf serum (FCS), 1 mM L-glutamine, and 50 *μ*g/mL penicillin/streptomycin (Gibco, Milan), in a humidified atmosphere containing 5% CO_2_ at 37°C. Cell lines were harvested in log-phase growth for all experiments, washed, and cultured to the appropriate concentration in RPMI 10% FCS ± TR at scalar concentrations (0.5–2 mg/mL).

Peripheral blood (PB) samples and bone marrow (BM) aspirate samples were obtained from normal donors and from 4 AML patients, respectively, all referred to our Hematology at Sapienza University of Rome, Italy. Written informed consent for *in vitro* studies was obtained from donors and patients in accordance with regulations and protocols sanctioned by the Human Subjects Committee of Helsinki and were approved by the Sapienza Institutional Review Board (protocol number 158/10 signed on February 18, 2010). MNC obtained from the PB of a normal volunteer donor and blast cells from the BM of AML patients were separated by layering on Ficoll-Hypaque density gradient (Lymphoprep; 1.007 g/mL). Cells used for *in vitro* studies were resuspended at a concentration of 1.0 × 106/mL, in RPMI 10% FCS ± TR at scalar concentrations (0.5–2 mg/mL). In particular, the circulating mononuclear cells obtained from healthy donors were placed in liquid culture ± scalar concentrations of TR in the absence and in the presence of a proliferative stimulus (PHA).

### 2.5. Cell Cycle and Apoptosis Analysis

Cell cycle distribution changes were evaluated using the Acridine Orange (AO) technique as previously described [23]. The percentage of cells in G0, G1, S, and G2 M was determined by measuring simultaneously the DNA and RNA total cellular content. The percentage of apoptotic cells was measured based on the decreased stainability of apoptotic elements in DNA green fluorescence (sub-G0/1 peak on DNA frequency histograms) coupled with a higher RNA red fluorescence (which is common to chromatin condensation); cell debris was excluded from the analysis on the basis of their forward light scatter properties. Cell cycle distribution was analyzed using the ModFit LT software (Verity Software House, Topsham, ME).

Induction of apoptosis was also assessed by measuring Annexin V binding to externalized phosphatidylserine, as previously described [24]. Briefly, cells were washed twice with PBS and resuspended in binding buffer (10 mM Hepes/NaOH pH 7.4, 140 mM NaCl, 2.5 mM CaCl_2_, Sigma Chemical Co.). FITC-conjugated Annexin V (Roche Diagnostic Corp., Indianapolis, Indiana, USA) was added at a final concentration of 1 *μ*g/mL. The mixture was incubated at room temperature for 15 min in the dark prior to flow cytometric analysis. Membrane integrity was simultaneously assessed by propidium iodide (PI, 0.25 *μ*g/mL) exclusion.

### 2.6. Analysis of Intracellular ROS Levels

The generation of ROS was measured by using DCFH-DA, a ROS-sensitive fluorescent probe. Briefly, OCI-AML3 cells were cultured for 24 h with different concentrations of TR. After exposure, cells were incubated with 10 mM DCFH-DA for 30 min at 37°C, then washed, resuspended in PBS, and immediately analyzed by flow cytometry. Results were expressed as mean fluorescence intensity (MFI) relative to that of control.

### 2.7. Statistical Analysis

The two-sided Student *t*-test was used to evaluate the significance of differences between groups. Results were expressed as the mean ± standard deviation (SD). Values of *p* < 0.05 were considered significant from a statistical point of view.

## 3. Results

### 3.1. Effects of TR on Leukemia Cell Lines

We started to evaluate the effects of TR on human myeloid (OCI-AML3) and lymphoid (Jurkat) leukemia cell lines exposed to scalar concentrations of TR (0.5–2 mg/mL). Cell aliquots were harvested at 24, 48, and 72 hours and analyzed for cell counts (trypan blue), cell cycle (flow cytometric analysis by AO), and apoptosis (flow cytometry assessment with AO, Annexin V).

**Figure 1:**
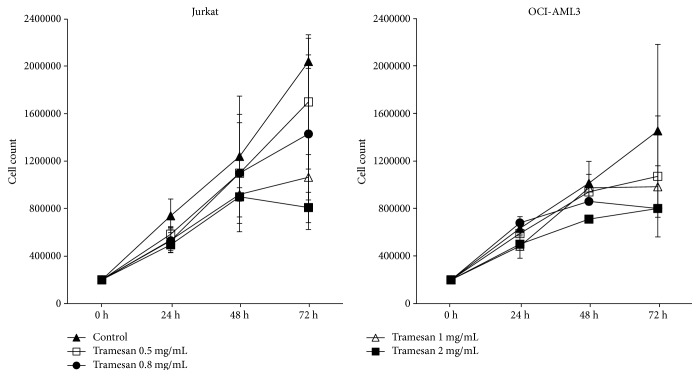
TR induces a dose-dependent reduction in cell count in hematopoietic cell lines. Jurkat and OCI-AML3 cells were exposed to increasing concentrations of TR for the indicated time points. Cell counts and viability were then assessed by trypan blue exclusion counting. Results are expressed as the average ± SD of seven independent experiments.

**Figure 2:**
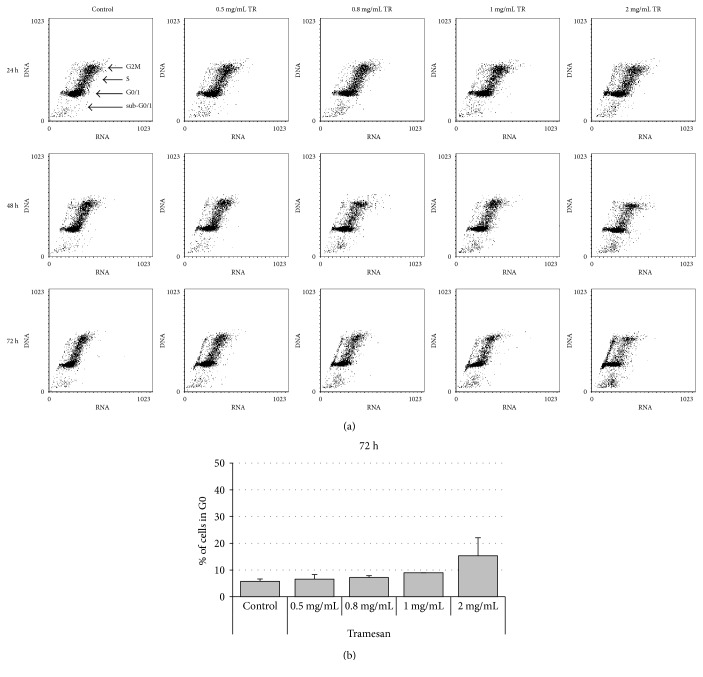
TR induces a dose-dependent increase of cells in G0 phase of cell cycle in hematopoietic cell lines. Jurkat cells were exposed to increasing concentrations of TR. Cell cycle changes were then evaluated by the AO technique. Results showed are representative of 7 independent experiments (a). Percentages of cells in G0 phase after 72 h of TR exposure are shown in (b).

Exposure to TR induced a dose- and time-dependent reduction in cell count in all hematopoietic cell lines studied (Figure 1). In fact, the effects were markedly evident at a dose of 1 mg/mL after 48 hours of liquid culture. The prolonged exposure to TR and increasing scalar concentrations of the molecule potentiated the effectiveness (Figure 1). Detection of cell cycle changes, measured at the same time points, however, showed that on leukemia cell lines TR was unable to affect cell cycle distribution and apoptosis. Only on Jurkat cell line it was observed, after 72 h of culture, a mild, not significant, dose-dependent increase of cells in G0 phase associated with a decrease of G1 and S compartment and with a nonsignificant increase of sub-G0/1 (Figure 2).

**Figure 3:**
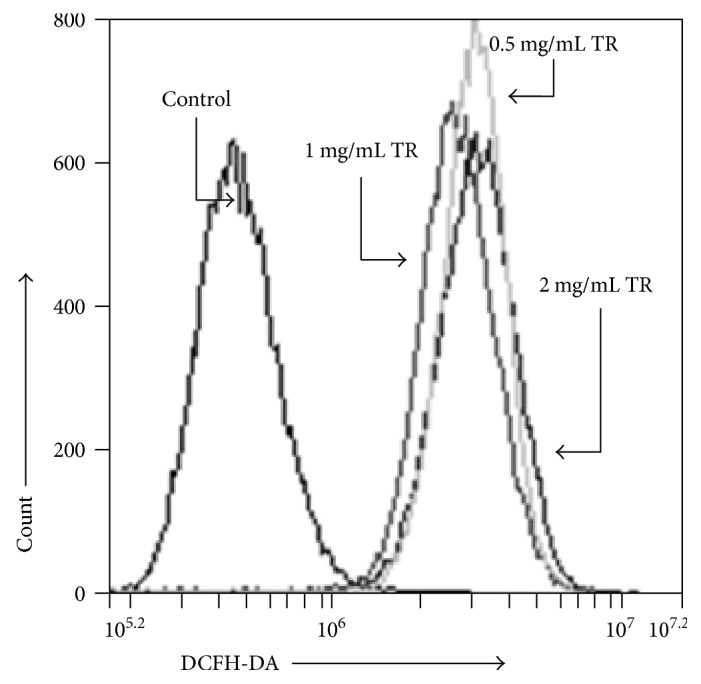
TR induced accumulation of ROS in hematopoietic cells line. Cells were exposed to increasing concentrations of TR. After 24 h, cells were stained with DCFH-DA for intracellular ROS level analysis.

Analysis of intracellular ROS levels in cells stained with DCFH-DA depicts (Figure 3) that TR induced accumulation of ROS as demonstrated by an increase of DCFH-DA MFI in OCI-AML3 treated with TR as compared to control: from MFI of 0.46 × 10^6^ (control) to MFI of 2.94 × 10^6^, 2.61 × 10^6^ and, 2.98 × 10^6^ in the presence of 0.5, 1, and 2 mg/mL TR, respectively.

### 3.2. Effects of TR on Primary Cells from AML Patients

**Figure 4:**
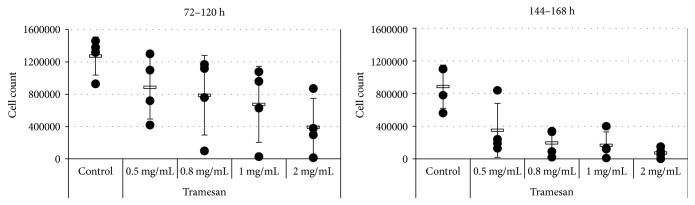
TR induces a dose- and time-dependent reduction in cell count in primary cells from AML patients. AML primary cells were exposed to the indicated concentrations of TR. Cell counts and viability were assessed for the individual sample after 72–120 h and 144–168 h by trypan blue exclusion.

We then examined the biological effects induced in vitro by TR on primary cells from 4 patients with newly diagnosed AML. Our results indicate that TR induced reduction in cell count and significant cytotoxic effects in a dose- and time-dependent fashion in all (4/4) samples studied. After 72–120 hours of culture, TR was able to exert a significant reduction in cell counts from 1272500 ± 235425 cells/mL, in the control condition, to 885000 ± 392386 (*p* = 0.06), 787500 ± 493381 (*p* = 0.04), 675000 ± 470213 (*p* = 0.02), and 391750 ± 356435 (*p* = 0.01) cells/mL in the presence of 0.5, 0.8, 1, and 2 mg/mL of TR, respectively (Figure 4).

**Figure 5:**
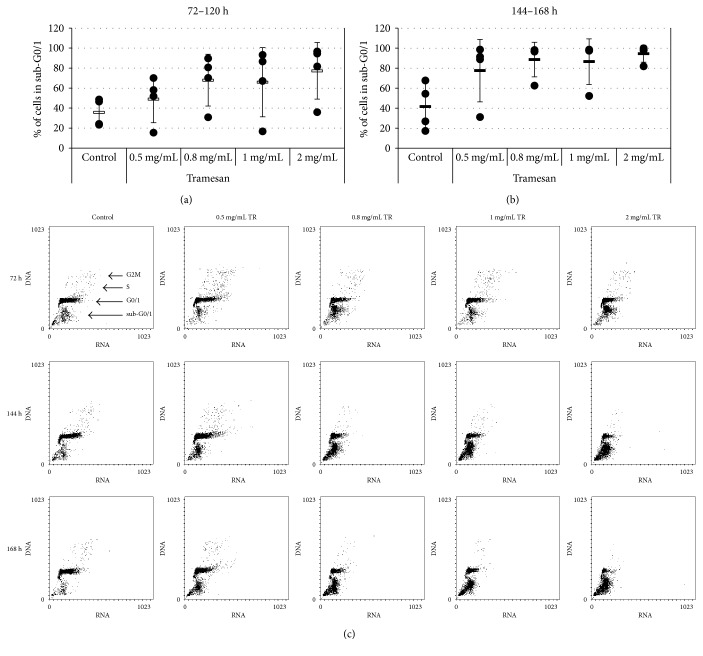
TR induces a dose- and time-dependent apoptosis in primary cells from AML. AML primary cells were exposed to the indicated concentrations of TR. Apoptosis induction was then evaluated by the AO technique. Percentages of sub-G0/1 cells are shown for the individual sample after 72–120 h (a) and 144–168 h (b) exposure to scalar concentrations of TR. A representative experiment is shown (c).

Notably, in primary AML samples, TR significantly increased apoptosis levels (sub-G0/1) from 35.7 ± 13.8%, in the control conditions, to 48.9% ± 23.5, 67.9% ± 25.9, 65.9% ± 34.6, and 77.2% ± 28.3 (*p* = 0.04) in the presence of 0.5, 0.8, 1, and 2 mg/mL of TR, respectively (Figure 5(a)). This effect was, however, even more marked at a prolonged exposure to the molecule (144–168 hours), reaching the statistical significance already at the lowest dose: from 41.6% ± 23.5, in the control condition, to 77.5% ± 31.1 (*p* = 0.04), 88.6% ± 17.3 (*p* = 0.01), 86.5% ± 22.8 (*p* = 0.01), and 94.5% ± 8.4 (*p* = 0.01), with 0.5, 0.8, 1, and 2 mg/mL of TR, respectively (Figures 5(b) and 5(c)).

### 3.3. Effects of TR on Normal Cells

**Figure 6:**
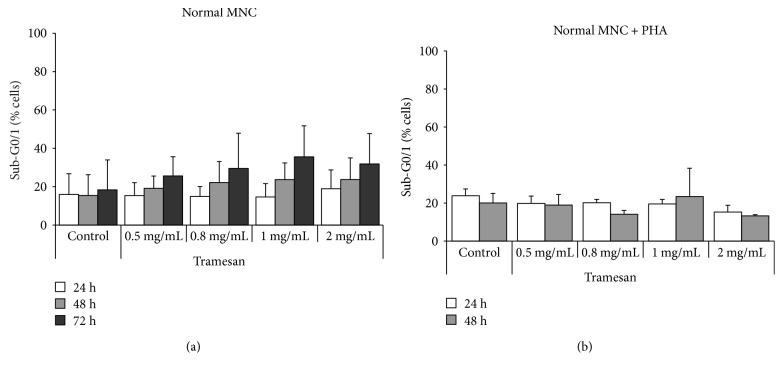
TR does not exert proapoptotic activity on normal MNC. Analysis of the levels of apoptosis (% sub-G0/1) in nonstimulated lympho-monocytes (a) and in those stimulated with PHA (b) and subsequently exposed to scalar concentrations of TR.

In order to analyze the activity of the compound on normal hematopoietic cells, we investigated the effects of TR on mononuclear cells obtained from PB of normal donors. The MNC were then exposed in liquid culture ± TR at scalar concentrations (0.5, 0.8, 1, and 2 mg/mL), in the absence and in the presence of a proliferative stimulus (PHA). The results obtained showed that TR was able to induce only a moderate, not statistically significant, proapoptotic activity, regardless of the dose: after 72 hours, apoptosis levels were 18.3% ± 15.6, in the control condition, and 25.65% ± 10.0, 29.5% ± 18.4, 35.5% ± 16.2, and 31.8% ± 15.8, with 0.5, 0.8, 1, and 2 mg/mL of TR, respectively (Figure 6(a)). These effects were not enhanced by the pretreatment with PHA: at 48 hours, apoptosis levels were 20.1% ± 5.0, in the control condition, and 18.9% ± 5.6, 14.1% ± 2.6, 23.4% ± 14.9, and 13.3% ± 1.0, with 0.5, 0.8, 1, and 2 mg/mL of TR, respectively (Figure 6(b)).

## 4. Discussion

The present study demonstrated that TR, a polysaccharide extract from *T. versicolor*, significantly suppresses cell growth of human leukemia cell lines from different ontogenesis and exerts a cytotoxic activity on primary cells from AML patients.

*T. versicolor* is one of the most commonly used medicinal mushrooms, and several extracts have already been investigated for their antineoplastic effects. The cytotoxic activity of PSP and PSK extract from *T. versicolor* has been reported in myeloid and lymphoid leukemia cell line [25, 26]. It was also demonstrated that an ethanol-water extract from wild *T. versicolor* exerted in vitro antiproliferative and cytotoxic effects against leukemia and lymphoma cell lines [26]. Moreover, the antioxidant effects of different molecular mass enzymatic hydrolysates from PSPs were described [27]. In addition, results from a phase 1 clinical trial proved that an orally administered preparation from the *T. versicolor* may improve immune response in women with breast cancer after standard chemotherapy and radiotherapy [14].

We demonstrated, for the first time at our knowledge, the *in vitro* cytotoxic activity of a polysaccharidic fraction purified from *T. versicolor* on primary AML cells, as shown by a time- and dose-dependent induction of apoptosis.

One of the fundamental hallmarks of cancer cells is their capacity to proliferate persistently, eluding cell cycle checkpoint controls and growth inhibitory signals [28, 29]. Moreover, the acquired resistance toward apoptosis represents another key hallmark of cancer. Hence, molecules capable to block the uncontrolled proliferation and/or to induce to cell death have been evermore considered very promising for therapeutic applications in cancers. Previous reports demonstrated that PSK induces *in vitro* cytotoxic activity in tumor cell lines, via arrest of cell cycle and induction of apoptosis [11, 15].

Data presented in our study demonstrated that TR causes in leukemia models a dose-dependent increase of cells in G0 phase and a decrease in both G1 and S phases. Nevertheless, lack of statistical significance argues in favor of a deceleration of cell cycle rather than a cell cycle arrest and apoptosis induction at these concentrations. The preclinical relevance of TR was showed on primary samples obtained from de novo AML patients. TR was indeed able to decrease the leukemic blast viability by significantly inducing apoptosis in all AML samples tested. By contrast, no significant cytotoxicity was observed on resting and activated normal MNC thus suggesting the existence of a therapeutic window in leukemia setting.

The direct antiproliferative effect of several fungal polysaccharides on cancer cells, caused by cell cycle arrest and apoptosis induction, has been also reported associated with oxidative stress [30, 31]. Here, in fact, we documented ROS accumulation in leukemia cell line. It was widely described that oxidants can contribute to cancer development by promoting cell mutation and growth [32]. Nevertheless, it was also reported that excessive oxidative stress can slow proliferation and induce cell death [32]. TR-induced cytotoxicity observed in primary AML samples could then be due to oxidative stress-induced cell death.

Although the literature seems to suggest the involvement of Toll-like receptors or of other cell surface receptors in the increased oxidative stress induced by fungal polysaccharides [33, 34], the exact mechanism remains to be clarified.

In conclusion, the cytotoxic preclinical activity of TR on primary leukemia cells, as compared to normal MNC obtained from healthy cells, makes TR an interesting bioactive compound among other antileukemic treatments and prompts further investigation aiming to elucidate its mechanism of action for a translational application.
